# Description of Hoskinsella mucinilytica gen. nov., sp. nov., a mucin-degrading isolate from human faeces, and reclassification of Amedibacillus hominis Abdugheni et al. 2023 as a later heterotypic synonym of Eubacterium hominis Liu et al. 2022 within the genus Hoskinsella gen. nov.

**DOI:** 10.1099/ijsem.0.007038

**Published:** 2026-01-20

**Authors:** Nicholas A. Pudlo, Gabriel Vasconcelos Pereira, Shaleni Singh, Rebecca M. Pollet, Thomas M. Schmidt, Philip B. Pope, Maria Chuvochina, Sabina Leanti La Rosa, Eric C. Martens

**Affiliations:** 1 Department of Microbiology and Immunology, University of Michigan, Ann Arbor, MI, USA; 2 Department of Internal Medicine, University of Michigan, Ann Arbor, MI 48109, USA; 3 Department of Ecology and Evolutionary Biology, University of Michigan, Ann Arbor, MI 48109, USA; 4 Faculty of Chemistry, Biotechnology and Food Science, Norwegian University of Life Sciences, 1432 Aas, Norway; 5 Faculty of Biosciences, Norwegian University of Life Sciences, 1432 Aas, Norway; 6 Centre for Microbiome Research, Faculty of Health, School of Biomedical Sciences, Queensland University of Technology, Translational Research Institute, Woolloongabba, Australia; 7 Australian Centre for Ecogenomics, School of Chemistry and Molecular Biosciences, The University of Queensland, Brisbane, QLD, Australia; 8 Leibniz Institute DSMZ-German Collection of Microorganisms and Cell Cultures, Braunschweig, Germany

**Keywords:** *Erysipelotrichaceae*, *Hoskinsella mucinilytica*, human faeces, mucin, taxonomy

## Abstract

Mucin-degrading bacteria are recognized as potential players in weakening of the colonic mucus barrier and increased susceptibility to pathogens in the human gut due to their use of mucus as a nutrient source, sometimes in the context of a fibre-deficient diet. Using intact porcine gastric mucin glycoprotein as the main nutrient source, we isolated a Gram-positive, non-motile, rod-shaped, obligately anaerobic bacterium (UGRO018_0423^T^) from faecal samples of a healthy adult. The strain grows with cells arranged in chains. Based on 16S rRNA gene sequence, the strain UGRO018_0423^T^ shared the highest similarity (96.94% and 96.75%) with *Eubacterium hominis* New-5^T^ and *Amedibacillus hominis* NSJ-176^T^, respectively. Phylogenomic analyses based on 120 concatenated phylogenetically informative protein markers (full-length protein and protein domain sequences) and examination of the placement within the Genome Taxonomy Database (GTDB) reference tree revealed that the strain UGRO018_0423^T^ is not affiliated with the genera *Eubacterium* or *Amedibacillus sensu stricto*, and placed UGRO018_0423^T^ within the family *Erysipelotrichaceae*. This also indicated the problematic taxonomic status of both *E. hominis* and *A. hominis* that were placed within the same genus as UGRO018_0423^T^ based on phylogenomic analyses. Genomic comparisons based on average nucleotide identity and digital DNA–DNA hybridization between UGRO018_0423^T^ and *E. hominis* New-5^T^ and *A. hominis* NSJ-176^T^ indicated that the strain UGRO018_0423^T^ represents a novel species. These results combined with further chemotaxonomic and phenotypic characterization indicated that strain UGRO018_0423^T^ (=DSM 116809^T^=ATCC TSD-466^T^) represents a novel species within a novel genus in the family *Erysipelotrichaceae*, for which the name *Hoskinsella mucinilytica* gen. nov., sp. nov. is proposed. In addition, based on genomic comparisons and phylogenomic analysis, *A. hominis* is reclassified as a later heterotypic synonym of *E. hominis,* and both species are transferred into the genus *Hoskinsella* gen. nov. with reclassification of *E. hominis* as *Hoskinsella hominis* comb. nov. and *A. hominis* as *Hoskinsella hominifaecis* nom. nov.

## Introduction

 The use of bacterial culturing to increase the catalogue of strains belonging to known species or to isolate novel or low-abundance taxa has provided substantial information about the individual members and collective function of the mammalian gut microbiota [1,3]. As nutritional and other competitive strategies that individual bacterial strains use to maintain colonization of the gastrointestinal tract continue to emerge, we gain increased understanding of the factors that dictate community dynamics [4,6]. One behaviour that is known to be advantageous for certain bacteria to compete in the gut is utilization of components of mucin glycoproteins, which function in part to form a protective barrier to shield host epithelial cells from direct contact with gut bacteria or their antigens [7,9]. Although numerous gut species, both commensal and pathogen, have been described for their mucin-degrading activities, much work still needs to be done to systematically uncover the complete inventory of micro-organisms capable of degrading mucin and which components (glycans, polypeptide backbone or both) in this very complex molecule are foraged by each species [10,16]. Towards this goal, we employed an isolation strategy using growth on solid medium containing intact porcine gastric mucin glycoprotein as the main nutrient source to isolate new mucin-degrading bacteria.

Our cultivation effort yielded an isolate capable of growing on porcine gastric mucin, UGRO018_0423^T^, that was determined to represent a novel species within the family *Erysipelotrichaceae*. A 16S rRNA analysis determined that the closest known relatives included *Eubacterium hominis* New-5^T^ (96.94%) and *Amedibacillus hominis* NSJ-176^T^ (96.75%) with closely related taxa also including the recently reclassified *Eubacterium* spp., *Amedibacterium intestinale* DSM 110575^T^ and *Amedibacillus dolichus* DSM 3991^T^ [2,17, 18]. However, genomic comparisons based on a genomic average nucleotide identity (ANI) by the National Center for Biotechnology Information (NCBI) and in this study indicated that *E. hominis* New-5^T^ and *A. hominis* NSJ-176^T^ should be assigned to the same species. Differences based on chemotaxonomic and phenotypic properties combined with phylogenomic analyses prompted us to propose a novel genus for the UGRO018_0423^T^ isolate and subsequent reclassification of *A. hominis* Abdugheni *et al*. 2023 as a later heterotypic synonym of *E. hominis* Liu *et al*. 2022 with the proposal of *Hoskinsella hominifaecis* comb. nov.

## Methods

### Isolation

The culture source was a faecal sample collected from a healthy volunteer who was a participant in a student cohort at the University of Michigan, Ann Arbor, MI, USA (42.278046 N −83.738220 W). All sample collection was approved by the Institutional Review Board of the University of Michigan Medical School (HUM00094242 and HUM00118951) in compliance with the Helsinki Declaration. Participants were part of an Authentic Research Section in an academic biology laboratory course at the University of Michigan (BIO173). Those who had taken antibiotics within 6 months or reported a history of irritable bowel syndrome, inflammatory bowel disease or colorectal cancer were excluded from the study. All participants or their legal guardian gave written consent to participate in the study. Participants ranged in age from 17 to 29 years old, with a median age of 19 years old. Because samples used for cultivation in this study had been deidentified, we did not retain exact age or sex information for the host source of UGRO018_0423^T^.

All bacterial growths were performed in an anaerobic chamber (Coy Manufacturing, Grass Lake, MI, USA) at 37 °C with an atmosphere of 85% N_2_, 10% H_2_ and 5% CO_2_. Human faecal samples were pre-enriched in a custom liquid chopped meat broth (CMB) medium [19] for 2 days with no antibiotics prior to streaking 10 µl onto a depleted media agar plate containing 1% porcine gastric mucin to isolate colonies [20]. Well-separated single colonies were streaked thrice on solid Brain Heart Infusion agar medium supplemented with 10% defibrinated horse blood (Quad Five) to ensure purity prior to growing a single colony in CMB. After isolation, strains were routinely cultivated in CMB or on Gifu Anaerobic agar plates (GAM), both of which are suitable rich media for bacterial growth.

### Mass spectrometry, microscopy and physiological analysis

Matrix-assisted laser desorption/ionization-time-of-flight Mass Spectrometry (MALDI-TOF MS) was performed on a MALDI Biotyper Sirius (BRUKER) according to the manufacturer’s protocols. Spectra were queried against the BRUKER Compass Explorer (v4.1.100) taxonomy database. The pH dependence analysis was performed by adjusting CMB between pH 4and 10 in 1.0 increments and incubating at 37 °C for 48 h. Microscopy, biochemical and cellular characterizations including cellular fatty acids, catalase, oxidase, salt, temperature, disc diffusion antibiotic testing and API-based (Analytical Profile Index) [50 CHB (6 days), 20A (17.5 h), Zym (6 h) and 32A (4 h)] methods were carried out anaerobically at 37 °C by DSMZ Services, Leibniz-Institut DSMZ – Deutsche Sammlung von Mikroorganismen und Zellkulturen GmbH, Braunschweig, Germany. For DSMZ analysis, the strain was cultivated on DSMZ medium 1203a (Fastidious Anaerobe Broth).

### DNA extraction and sequencing

DNA was extracted using phenol:chloroform (1:1) with bead-beating. The 16S rDNA sequencing was performed with universal primers 8F (5′-AGAGTTTGATCCTGGCTCAG-3′) and 1492R (5′-GGTTACCTTGTTACGACTT-3′). Illumina short-read sequencing was performed at the Microbial Genome Sequencing Center (MiGS; Pittsburgh, PA, USA; now SeqCenter). In addition, long-read sequencing was performed following Oxford Nanopore’s rapid genomic DNA barcoding protocol (SQK-LSK110) using a MinION sequencing platform (Oxford Nanopore Technologies) equipped with an R9.4 flow cell. The sequencer was controlled by the MinKNOW software v3.6.5, followed by base calling using Guppy v3.2.10 in ‘fast’ mode. Illumina short reads were processed using Trimmomatic v0.39 with --min_length 36 and --keep_percent 90 parameters. For the Nanopore long reads, the adapters were trimmed using PoreChop v0.2.4. The trimmed reads were used to construct a hybrid genome assembly using unicycler v0.4.8, polished with Pilon v1.23, and annotated using Prokka v1.11.

### Phylogenetic reconstructions by 16S rRNA sequence

The 16S rRNA sequence phylogenetic classification was generated by aligning representative related species matching >90% identity from NCBI blastn (Basic Local Alignment Search Tool nucleotide) searches. Sequence alignments were performed in the MUltiple Sequence Comparison by Log-Expectation (muscle; https://www.ebi.ac.uk/jdispatcher/msa) dispatcher from the European Molecular Biology Laboratory-European Bioinformatics Institute (EMBL-EBI) and exported as ClustalW files. The output file was converted in the Molecular Evolutionary Genetic Analysis software (MEGAX) to a mega file. A neighbour-joining and maximum-likelihood phylogenies were created using default parameters with a 1000- and 100-resampling bootstrap analysis, respectively.

### Taxonomic classification and phylogenetic reconstructions by genomic sequences

The final taxonomic assignment of isolate UGRO018_0423^T^ was achieved using a combination of methods, including calculation of 16S rRNA gene sequence identities, overall genome relatedness indices [digital DNA–DNA hybridization (dDDH), ANI and average amino acid identity (AAI)] and phylogenomic reconstructions. Values of >98.7% and 94.5–98.7% 16S rRNA gene sequence identity were used as an indication for the same species or genus, respectively [21]. ANI values <95% and dDDH values <70% were considered as an indication for separate species [22]. A threshold of 95% and 65% AAI value supported the status of novel species or genus, respectively [23]. 16S rRNA gene sequence identity against all taxonomically classified taxa of the Integrated Microbial Genomes and Microbiomes (IMG/M) database was determined by blastn [24]. ANI values were calculated using FastANI [25] and the ANI calculator tool available at http://enve-omics.ce.gatech.edu/ani/index. AAI values were determined using the AAI calculator tool available at https://enveomics.scigap.org/. The dDDH was calculated using the Genome-to-Genome Distance Calculator 2.0 (GGDC), a web service available at http://ggdc.dsmz.de.

Phylogenetic classification was first determined using the workflow in GTDB-Tk v2.4.0 [26], followed by the *de novo* tree reconstructions with data from Genome Taxonomy Database (GTDB) release 09-RS220. Based on genome relatedness results, 78 genomes designated by GTDB as species representatives of the type strains from the families *Eubacteriaceae* (*n*=12), *Erysipelotrichaceae* (*n*=46) and order *Halanaerobiales* (*n*=20; used as an outgroup) were obtained from the NCBI public repository. All genomes were of high quality, with more than 90% completeness and less than 5% contamination, except for one genome (*Lactimicrobium massiliense* P4301^T^) with 86% completeness that was still included in the classification. The preliminary *de novo* tree was inferred using a multiple sequence alignment (MSA) of 120 concatenated bacterial marker sequences (bac120; phylogenetically informative conserved single copy full-length protein and protein domain sequences; https://data.ace.uq.edu.au/public/gtdb/data/releases/latest/) using FastTree v2.1.10 with default parameters in GTDB-Tk v2.4.0. To obtain better resolution, the same MSA was subsequently used to infer a maximum-likelihood tree using IQ-TREE v2.1.2 (https://iqtree.github.io/) with mixture model ‘-m LG+ C10 + F + G’ and ultrafast bootstrap approximation. The tree was visualized using iTOL v6 [27].

## Results and discussion

In an attempt to generate an expanded catalogue of gut bacteria with mucin-degrading capabilities, we employed a previously effective selection approach based on a single carbon source [28]. To this aim, a depleted agar medium containing unmodified porcine gastric mucin was used to isolate 199 morphologically ‘unique’ colonies from 86 faecal samples via the streak plate method. Upon de-replication and Sanger sequencing the 16S rRNA gene of the isolates with one sequencing read, there was a single isolate, UGRO018_0423^T^, that had low 16S rRNA similarity (96.94%), at the time of sequencing, to existing sequences in the NCBI database. Similarly, the MALDI-TOF MS spectrum analysis did not match a known spectrum in the commercially available BRUKER database. Thus, we sequenced the genome of this isolate and were able to obtain a full-length 16S rRNA gene sequence that was still <97% nucleotide identity to known sequences in the family *Erysipelotrichaceae*, supporting the idea that this was a newly isolated species from the human gut microbiota. UGRO018_0423^T^ displayed growth on intact porcine gastric mucin glycoprotein as well as chemically purified *O*-linked glycans as sole energy sources but not on a carbohydrate-free media control [29]. Cultures of UGRO018_0423^T^ increased in optical density (Ab600) to 0.28±0.017 when grown in media containing purified porcine gastric mucin (1% w/v) as the sole carbohydrate source. Separate cultures with purified gastric mucin *O*-glycans as the sole carbohydrate source (3% w/v) increased to an Ab600 0.76±0.029. In both these experiments, negative control cultures using the same medium without any added carbohydrate only increased to 0.044±0.001 (the values listed for gastric mucin and purified *O*-glycans have this background turbidity subtracted from the average growth increase reported, with variation representing standard deviation of the mean, *n*=6 per growth condition).

**Fig. 1.**
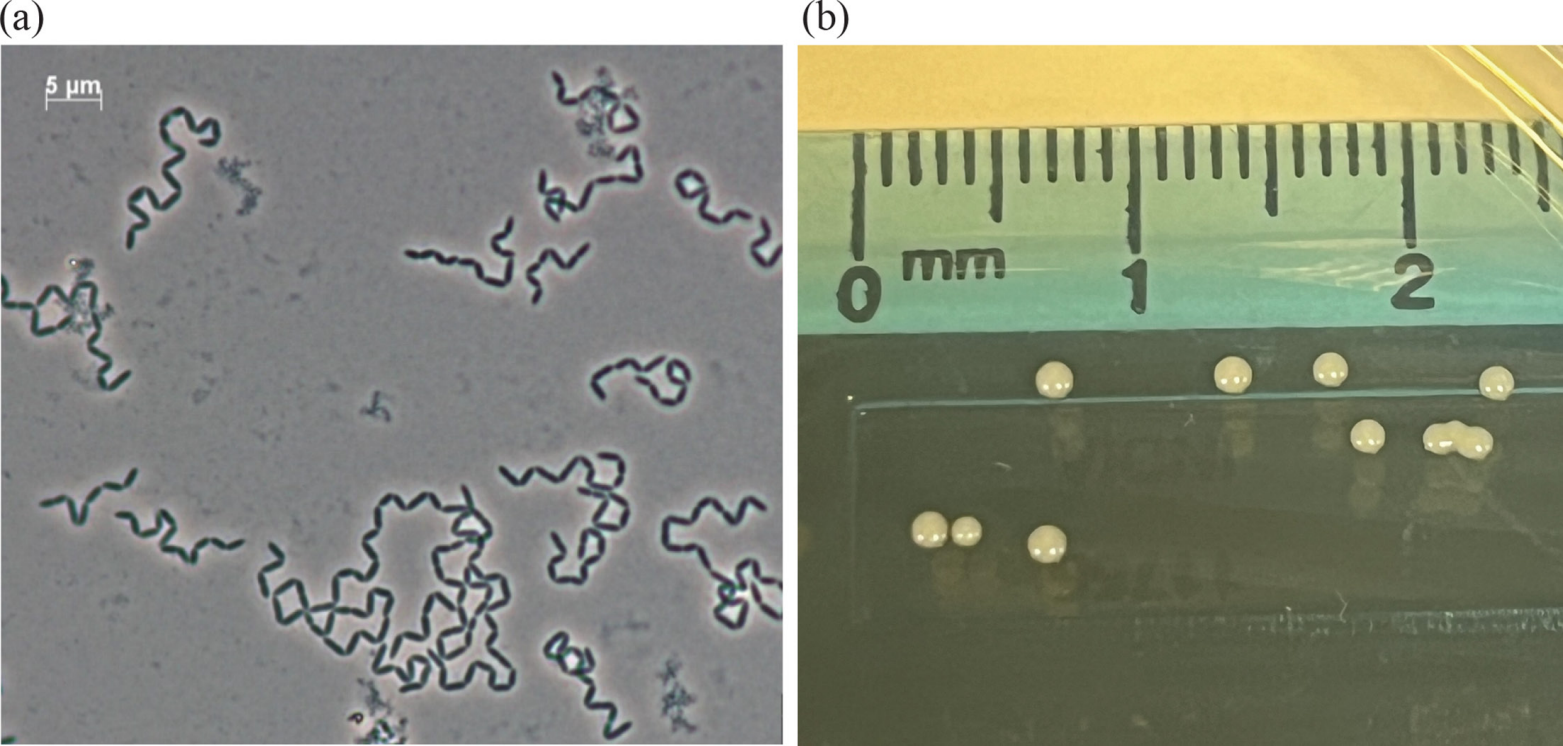
(**a**) Phase-contrast micrograph of UGRO018_0423^T^ and (**b**) colony morphology on GAM agar at 48 h.

It was determined that UGRO018_0423^T^ was a catalase- and oxidase-negative obligate anaerobe that stains Gram-positive and was non-motile. The cells are ~2–3 µm rods that form chains (Fig. 1a). Colonies grown on GAM agar after 2 days at 37 °C display 1–1.5 mm diameter and were round, creamy white, smooth and raised (Fig. 1b). Cells could tolerate salinity in the presence of 0–2% (w/v) NaCl and a pH between 6 and 9 (optimum 7.0), and growth occurred between 15 and 40 °C (optimal 35–37 °C).

The API ZYM kit positively detected leucine-arylamidase, cystine-arylamidase and naphthol-AS-BI-phosphohydrolase activity, but not for alkaline phosphatase, esterase, esterase lipase, lipase, valine-arylamidase, trypsin, chymotrypsin, acid phosphatase, *α*-galactosidase, *β*-galactosidase, *β*-glucuronidase, *α*-glucosidase, *β*-glucosidase, *β*-*N*-acetyl-glucosaminidase, *α*-mannosidase or *α*-fucosidase.

Acid production from the API 20A was detected from mannitol (weak), sucrose, maltose, salicin, cellobiose, mannose, melezitose, raffinose, trehalose and l-rhamnose, but not from glucose, lactose, xylose, l-arabinose, glycerol, mannose or sorbitol. There was hydrolysis of aesculin, but not for gelatin. No indole or urease was detected.

The API rapid 32A showed arginine dihydrolase (weak), *α*-glucosidase, *β*-glucosidase, *N*-acetyl-*β*-glucosaminidase, arginine arylamidase, phenylalanine arylamidase, leucine arylamidase, tyrosine arylamidase, alanine arylamidase, histidine arylamidase, glutamyl-glutamic acid arylamidase and serine arylamidase activity. No activity was detected for *α*-galactosidase, *β*-galactosidase, *β*-galactosidase 6-phosphate, *α*-arabinosidase, *β*-glucuronidase, mannose fermentation, raffinose fermentation, glutamic acid decarboxylase, *α*-fucosidase, nitrate reduction, indole production, alkaline phosphatase, proline arylamidase, leucyl-glycine arylamidase, pyroglutamic acid arylamidase or glycine arylamidase.

The API 50 CHB test showed acid in the presence of glucose (weak), fructose, rhamnose, mannitol (weak), *α*-Me-d-glucoside, *N*-acetylglucosamine, amygdalin, arbutin, aesculin, salicin, cellobiose, melibiose, sucrose, trehalose, melizitose, raffinose, gentibiose and d-turanose, but not glycerol, erythritol, d-arabinose, l-arabinose, ribose, d-xylose, l-xylose, adonitol, *β*-Me-d-xyloside, galactose, mannose, sorbose, dulcitol, inositol, sorbitol, *α*-Me-d-mannoside, maltose, lactose, inulin, starch, glycogen, xylitol, d-lyxose, d-tagatose, d-fucose, l-fucose, d-arabitol, l-arabitol, gluconate, 2-ketogluconate and 5-ketogluconate. 

Major fatty acids detected are C_16:0_ fatty acid methyl ester (12.4%), C_16:0_ dimethyl acetal (DMA) (18.9%) and C_18:1_ *ω*9c (22.4%). The UGRO018_0423^T^ cellular characteristics and fatty acid comparisons between related type strains are represented in Tables1  2, respectively.

**Table 1.** Characteristics of strain UGRO018_0423^T^ with related type strains from four different genera Strains: 1, UGRO018_0423^T^; 2, *A. hominis* NSJ-176^T^; 3, *A. intestinale* DSM 110575^T^; 4, *A. dolichus* DSM 3991^T^; 5, *Clostridium innocuum* ATCC14501^T^; 6, *Longicatena caecimuris* DSM 29481^T^. +, Positive; −, negative; w, weak; nd, not determined. All data other than strain UGRO018_0423^T^ is from ^Liu *et al*. [2] and Abdugheni *et al*. [17] or *Ikeyama *et al*. [18].

Characteristic	1	2^	3*	4*	5*	6*
Colony morphology	Convex	Convex	Umbilicate	Convex	Convex	Convex
Aesculin hydrolysis	+	+	+	−	+	− (+)
**Acid produced from:**						
Cellobiose	+	+	+	−	+	−
Glucose	−	+	+	−	+	−
Maltose	+	+	+	−	−	−
d-Mannitol	w	w	+	−	−	−
d-Mannose	−	+	+	−	+	−
Salicin	+	nd	+	−	+	−
Trehalose	+	+	+	−	+	−
**Enzyme activities:**						
*N*-Acetyl-*β*-glucosaminidase	+/−	−	+	+	−	−
Acid phosphatase	w	+	+	+	+	w
Alkaline phosphatase	w	−	+	+	w	−
Esterase	−	+	+	w	w	w
Esterase lipase	w	+	+	w	w	w
*α*-Glucosidase	w	−	w	−	−	−
*β*-Glucosidase	w	−	−	w	−	−
Naphthol-AS-BI-phosphohydrolase	+	+	+	+	+	w
Major cellular fatty acids (in descending order)	C_18:1_ *ω*9c C_16:0_ DMA C_16:0_	C_18:1_ *ω*9c C_16:0_ C_18:0_	C_18:1_ *ω*9c C_18:1_ *ω*9c DMA C_16:0_ DMA	C_18:1_ *ω*9c C_16:0_	C_16:0_ C_18:1_ *ω*9c	C_18:1_ *ω*9c

**Table 2.** Percentages of cellular fatty acids for strain UGRO018_0423^T^ and related type strains from four different genera Strains: 1, UGRO018_0423^T^; 2, *A. hominis* NSJ-176^T^; 3, *A. intestinale* DSM 110575^T^; 4, *A. dolichus* DSM 3991^T^; 5, *Longicatena ceacimuris* DSM 29481^T^; 6, *Clostridium innocuum* ATCC14501^T^. ALDE, aldehyde; −, not detected/reported. Data other than strain UGRO018_0423^T^ is from (a) Abdugheni *et al*. [17], (b) Ikeyama *et al*. [18], (c) Lagkouvardos *et al*. [31] and (d) Ghimire *et al*. [32].

Fatty acid	1	2 (a)	3 (b)	4 (b)	5 (c)	6 (d)
C_12:0_	1.2	–	–	–	–	3.28
C_14:0_	5.7	–	7.8	3.3	–	8.85
C_16:0_	**12.4**	**16.44**	9.6	**14.0**	4.6	**23.7**
C_17:0_	–	4.93	–	–	–	–
C_18:0_	6.2	**14.57**	5.7	5.3	1.9	**10.56**
C_14:0_ ALDE	0.4	–	–	–	–	–
C_14:0_ DMA	1.0	–	–	–	–	–
C_14:1_* ω7*c	0.7	–	–	–	–	–
C_16:0_ ALDE	3.0	–	–	–	–	2.78
C_16:0_ DMA	**18.9**	–	**11.1**	–	–	9.95
C_16:1_* ω7*c	4.4	–	–	–	–	**10.59**
C_16:1_* ω9*c	7.9	2.76	–	–	–	6.51
C_18:0_ ALDE	1.8	–	–	–	–	–
C_18:0_ DMA	9.9	–	–	–	–	2.83
C_18:1_ DMA	0.5	–	–	–	–	–
C_18:1_ *ω*9c DMA	–	–	**17.3**	–	–	–
C_18:1_* ω*7c	–	–	–	–	5.7	3.08
C_18:1_* ω*9c	**22.4**	**25.77**	**46.3**	**65.3**	**77.4**	**14.64**
C_18:1_* ω*11c	3.8	–	–	–	–	–
C_18:2_* ω*6,9c	–	–	7.8	2.7	–	–

Disc diffusion measurements showed no inhibition in response to the following antibiotics (unit values in parentheses are μg for all antibiotics except polymyxin, which is international units): aztreonam (30), ceftazidime (10), cefiderocol (30), amikacin (30), gentamicin (30), polymyxin b (300), colistin sulphate (10), kanamycin (30), rifampicin (5) and trimethoprim-sulfamethoxazole (10). The following zones of inhibition measured by diameter were observed for the following antibiotic concentrations (first unit values in parentheses are μg of antibiotic, second unit values are mm of inhibition zone): ampicillin (10; 26), oxacillin (5; 6), penicillin g (6; 24), ticarcillin (75; 34), cefotaxime (30; 30), ceftriaxone (30; 24), piperacillin/tazobactam (110; 26), imipenem (10; 24), meropenem (10; 22), ciprofloxacin (5; 16), levofloxacin (5; 18), moxifloxacin (5; 20), ofloxacin (5; 13), tetracycline (30; 22), tigecycline (15; 18), teicoplanin (30; 14-20), vancomycin (30; 16), clindamycin (10; 22-24), erythromycin (15; 20), fosfomycin (50; 22), chloramphenicol (30; 20), linezolid (10; 20), nitrofurantoin (100; 22) and quinupristin/dalfopristin (15; 26).

The genome of UGRO018_0423^T^ was found to have a size of 3.73 Mb, encompassing a total of 3,512 genes with a 35 mol% G+C content. Of the annotated genes, 3,466 contained a coding sequence with the remainder as rRNA (3), tRNA (42) and tmRNA (1) genes. Genome content comparisons to closely related taxa are summarized in Table 3.

**Table 3.** Genome comparison between the UGRO018_0423^T^ and related type strains from four different genera

Organism/strain	Size (Mb)	G+C (mol%)	CDS (total)	16S rRNA copies	tRNA	NCBI RefSeq assembly
UGRO018_0423^T^	3.7	35	3,466	3	42	GCF_045223415.1
*A. hominis* NSJ-176^T^	4.4	35	4,214	4	45	GCF_022487425.1
*Longicatena caecimuris* DSM 29481^T^	2.9	38	2,703	3	43	GCF_004341945.1
*Clostridium innocuum* ATCC 14501^T^	4.7	44	4,525	5	48	GCF_012317185.1
*A. intestinale* DSM 110575^T^	2.5	34	2,381	6	66	GCF_010537335.1
*A. dolichus* DSM 3991^T^	2.2	38	2,166	2	41	GCF_000154285.1

**Fig. 2.**
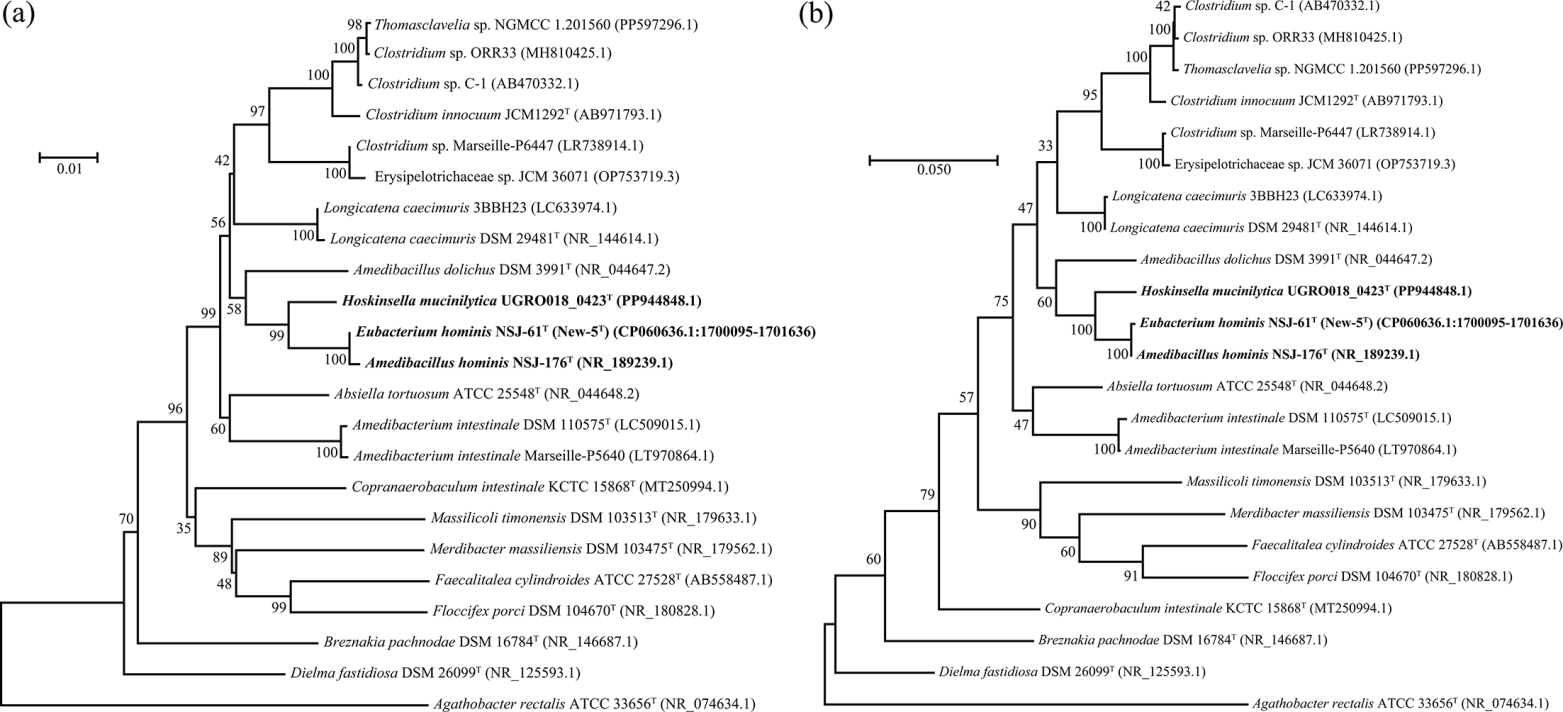
Phylogenetic trees generated from 16S rRNA gene sequences to show the relationship between the strain UGRO018_0423^T^ and related strains from NCBI blastn results. (**a**) A neighbour-joining phylogeny based on 1,000 replicates. (**b**) A maximum-likelihood phylogeny based on 100 replicates. All type strain sequences with >90% similarity to the UGRO018_0423^T^ strain were used for tree generation. Some redundant (*Longicatena* and *Amedibacterium* spp.) or unclassified species strains (e.g. *Clostridium* sp.) were also used to either confirm validity of phylogenetic constructions or determine relatedness of unclassified species designations, respectively. GenBank accession numbers are shown in parentheses. All bootstrap values are shown at branch nodes. Bar size indicates substitutions per nucleotide position.

Based on 16S rRNA gene sequence analysis, the closest sequence to UGRO018_0423^T^ was *E. hominis* strain New-5^T^, sharing 96.94% sequence similarity, suggesting that the two bacteria likely belong to different species. A 16S rRNA phylogenetic analysis of isolates with >90% 16S rRNA sequence similarity to UGRO018_0423^T^ suggested that another isolate, *A. hominis* NSJ-176^T^, which had 96.75% similarity was most closely related to *E. hominis* strain New-5^T^ with 99.86% similarity, and both were the nearest neighbours to UGRO018_0423^T^ (Fig. 2). To further investigate relatedness amongst these strains, we employed a more comprehensive genomic approach. The status of distinct species for UGRO018_0423^T^ was confirmed by ANI (79.36%) and dDDH (20.1%) values determined via pairwise comparison with *E. hominis* strain New-5^T^ (NSJ-61^T^). It is worth noting that the *E. hominis* strain New-5^T^ (GenBank assembly GCA_014337235.1) was phylogenetically placed in the genus *Eubacterium* (phylum *Bacillota*, family *Eubacteriaceae*) by Liu *et al*. [2,30], based on ANI and dDDH values best matching the genome of *Eubacterium dolichum* DSM 3991^T^ (NZ_ABAW00000000.2). However, *E. dolichum* DSM 3991^T^ was recently reclassified as *A. dolichus* [18], a member of the family *Erysipelotrichaceae,* thus also highlighting the distinct phylogenetic position of strain New-5^T^ from the rest of the other species within the genus *Eubacterium*. Consequently, the taxonomic proposal published by Liu *et al*. [2,30] for *E. hominis* strain New-5^T^ (NSJ-61^T^) suggests a need for reconsideration, either to reclassify this organism as a member of *Amedibacillus* or as a member of a novel genus.

**Fig. 3.**
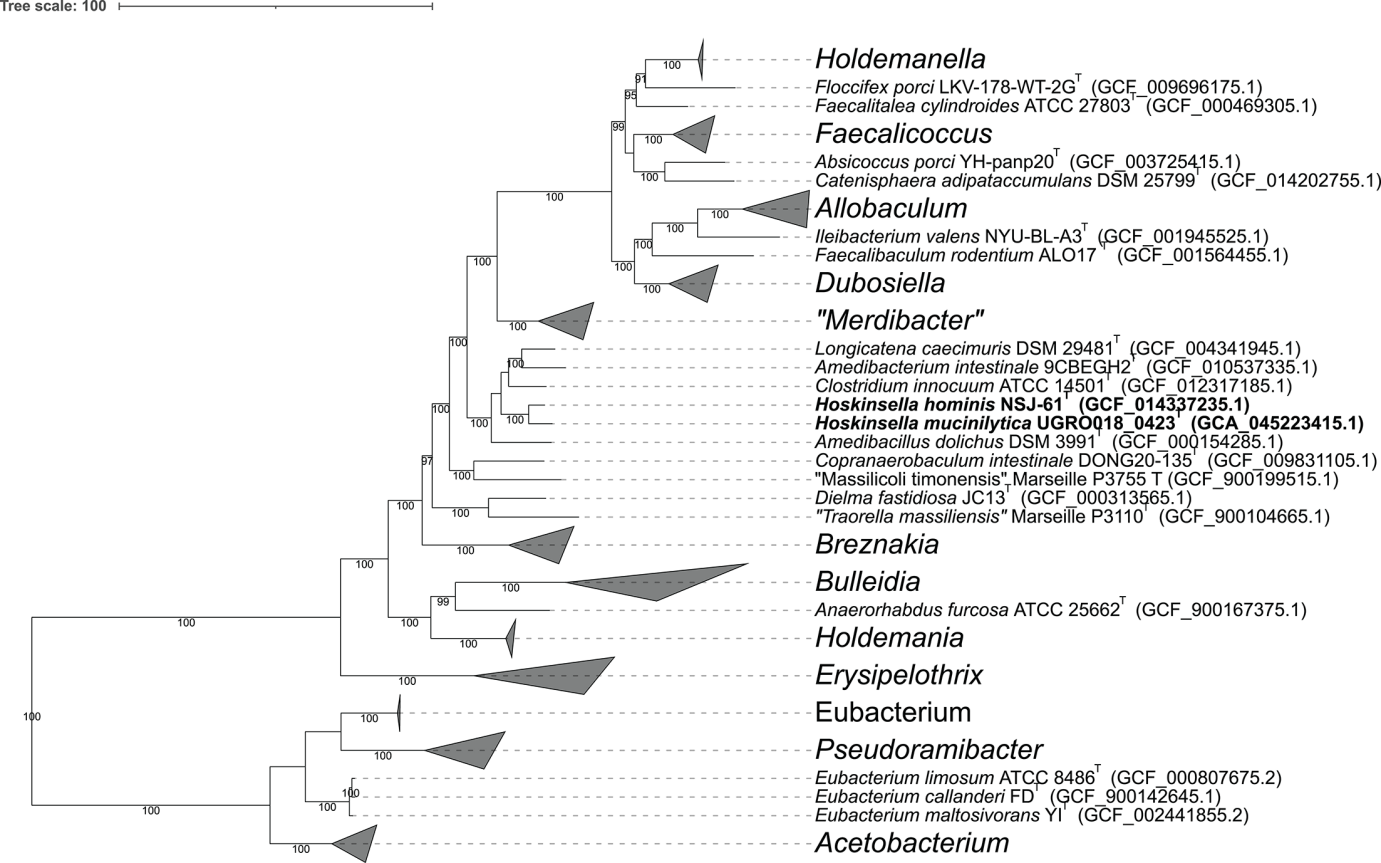
Phylogenomic maximum-likelihood tree showing the relationships of the study strain UGRO018_0423^T^ with 12 *Eubacteriaceae* and 46 *Erysipelotrichaceae* type strains within the phylum *Bacillota*. The tree is based on the alignment of 120 protein markers from GTDB genome representatives defined in GTDB 09-RS220 (https://gtdb.ecogenomic.org/). The tree was inferred using IQ-TREE, with ultrafast bootstrap values displayed below the nodes, and rooted on members of the order *Halanaerobiales* (not shown for illustrative purposes). Taxonomic assignments are based on GTDB 09-RS220.

The genome-based phylogenomic tree also revealed that strain UGRO018_0423^T^ formed a well-supported cluster with *E. hominis* New-5^T^ (NSJ-61^T^) in the family *Erysipelotrichaceae* (Fig. 3), and both species belong to the same genus based on the GTDB-Tk results (g__Eubacterium_P). Furthermore, based on the GTDB release 09-RS220, genomes from *E. hominis* New-5^T^ and *A. hominis* NSJ-176^T^ are classified as members of the same species cluster (s__Eubacterium_P hominis). This was also confirmed in the present study based on ANI and DDH values (99.73%; 98.3%) among the two species. The AAI value between *E. hominis* New-5^T^ and *A. hominis* NSJ-176^T^ is 99.14%; between *E. hominis* New-5^T^ and UGRO018_0423^T^ is 71.64%; and between *A. hominis* NSJ-176^T^ and UGRO018_0423^T^ is 72.5%. All these AAI values are within the genus cutoff. Therefore, the results from the phylogenomic analyses and genomic relatedness strongly support the conclusion that the strain UGRO018_0423^T^ represents a new species within the same genus as *E. hominis* and *A. hominis*. Additionally, this study clarifies the taxonomic position of the two latter species that were initially erroneously classified as members of the genera *Eubacterium* and *Amedibacillus* [2,17]*,* respectively, and should be treated as heterotypic synonyms.

Based on the evidence from the phylogenetic analyses and chemotaxonomic and physiological features, strain UGRO018_0423^T^ belongs to a novel distinct species within a new genus designated as *Hoskinsella mucinilytica* gen. nov. sp. nov. In addition, we propose the reclassification of *A. hominis* Abdugheni *et al*. 2023 as a later heterotypic synonym of *E. hominis* Liu *et al*. 2022 and the transfer of *E. hominis* into the genus *Hoskinsella* gen. nov. as *Hoskinsella hominis* comb. nov. and *A. hominis* as *H. hominifaecis* nom. nov., where the two species are treated as heterotypic synonyms.

## Description of *Hoskinsella* gen. nov.

*Hoskinsella* [Hos.kins.el’la. N.L. gen. n. *Hoskinsella*, named after Lansing C. Hoskins (Professor of Medicine at Case Western Reserve University, USA) for his pioneering research on mucin foraging bacteria from the human gut microbiota].

Gram-stain-positive, obligately anaerobic, non-motile, rod-shaped cells isolated from human faeces. Cells are ~2–3 µm in size with growth optima of 37 °C and pH 7.0. The DNA G+C content is between 35 and 36 mol%. Cells metabolize cellobiose and maltose. The predominant cellular fatty acid is C_18:1_ *ω*9c. The genus is defined based on its distinct phylogenetic position in the concatenated protein tree as a member of the family *Erysipelotrichaceae*. The genus contains two species, *H. mucinilytica* and *Hoskinsella hominis*, which were isolated from human faeces. The type species is *H. mucinilytica*.

## Description of *Hoskinsella mucinilytica* sp. nov.

*Hoskinsella mucinilytica* (mu.ci.ni.ly’ti.ca. N.L. neut. n. *mucinum*, mucin, Gr. masc. adj. *lytiko*s, dissolving, N.L. fem. adj. *mucinilytica*, mucin-dissolving).

Displays the following characteristics and activities in addition to those in the genus description: optimal growth occurs at 37 °C with a pH of 7.0 in broth containing <2% (w/v) NaCl (optimum, 1.0%). Colonies on GAM agar after 2 days (anaerobic, 37 °C) are ~1–1.5 mm with a round, creamy white, smooth and raised morphology. Leucine-arylamidase, cystine-arylamidase and naphthol-AS-BI-phosphohydrolase are detected. Acid is produced in 18 h from mannitol (weak), sucrose, maltose, salicin, cellobiose, mannose, melezitose, raffinose, trehalose and l-rhamnose. There is hydrolysis of aesculin. No indole or urease is detected. Activity is detected for arginine dihydrolase (weak), *α*-glucosidase, *β*-glucosidase, *N*-acetyl-*β*-glucosaminidase (variable), arginine arylamidase, phenylalanine arylamidase, leucine arylamidase, tyrosine arylamidase, alanine arylamidase, histidine arylamidase, glutamyl-glutamic acid arylamidase and serine arylamidase. Acid is detected at 6 days in the presence of glucose (weak), fructose, rhamnose, mannitol (weak), *α*-Me-d-glucoside, *N*-acetylglucosamine, amygdalin, arbutin, aesculin, salicin, cellobiose, melibiose, sucrose, trehalose, melizitose, raffinose, gentibiose and d-turanose. Predominant cellular fatty acids are C_18:1_ *ω*9c, C_16:0_ DMA and C_16:0_. Cells are susceptible to ampicillin, oxacillin, penicillin, ticarcillin, cefotaxime, ceftriaxone, piperacillin/tazobactam, imipenem, meropenem, ciprofloxacin, levofloxacin, moxifloxacin, ofloxacin, tetracycline, tigecycline, teicoplanin, vancomycin, clindamycin, erythromycin, fosfomycin, chloramphenicol, linezolid, nitrofurantoin and quinupristin/dalfopristin. Cells are resistant to aztreonam, ceftazidime, cefiderocol, amikacin, gentamicin, polymyxin b, colistin sulphate, kanamycin, rifampicin and trimethoprim-sulfamethoxazole.

The type strain, UGRO018_0423^T^ (=ATCC TSD-466^T^=DSM 116809^T^), was isolated from a faecal sample of a healthy adult in the USA. The genome size of the type strain is 3.73 Mb and the DNA G+C content is 35 mol%. The GenBank accession numbers for the 16S rRNA gene sequence and the whole-genome sequence of the strain are PP944848 and GCA_045223415.1, respectively.

## Reclassification of *Amedibacillus hominis* Abdugheni *et al*. 2023 as a later heterotypic synonym of *Eubacterium hominis* Liu *et al*. 2022

A comparison between the genomes from the type strains of *E. hominis* (GCF_014337235.1) and *A. hominis* (GCA_022487425.1) revealed ANI and dDDH values of 99.73% and 98.3%, respectively. This finding strongly indicates that the two species should be considered as conspecific. The genomic similarities are also supported by phenotypic characteristics of these species that include being rod-shaped obligate anaerobes that grow as circular, white and smooth colonies on mGAM agar plates at 37 °C. Cells are ~0.5–0.8 µm wide and ~1–2 µm long with an optimum growth pH at ~7.0. The two isolates also share the ability to metabolize at least 19 simple sugars tested, including fructose, gentiobiose, *α*-d-glucose, maltose, 3-methyl-d-glucose, palatinose, d-raffinose, glyoxylic acid, d-cellobiose, l-fucose, d-galactose, d-galacturonic acid, d-gluconic acid, glucose-6-phosphate, *α*-d-lactose, lactulose, maltotriose, d-mannitol, d-mannose and d-melibiose. Consequently, we propose *A. hominis* Abdugheni *et al*. 2023 as a later heterotypic synonym of *E. hominis* Liu *et al*. 2022.

## Description of *Hoskinsella hominis* comb. nov.

*Hoskinsella hominis* (ho'mi.nis. L. gen. n. *hominis*, from a human being).

Basonym: *Eubacterium hominis* Liu *et al*. 2022

The description is the same as that for *E. hominis* given by Liu *et al*. [2]. The DNA G+C content of the type strain is 35 mol%. The genome sequence accession number is GCA_014337235.1. The species has the following later heterotypic synonyms: *A. hominis* NSJ-176^T^ (=CGMCC 1.17933^T^=KCTC 25355^T^) and *H. hominifaecis* NSJ-176^T^ (=CGMCC 1.17933^T^=KCTC 25355^T^). The type strain is New-5^T^ (=NSJ-61^T^=CGMCC 1.32837^T^=KCTC 15860^T^), isolated from human faeces.

## Description of *Hoskinsella hominifaecis* nom. nov.

*Hoskinsella hominifaecis* (ho.mi.ni.fae’cis. L. masc. n. *homo*, a human being; L. fem. n. *faex*, the dregs, faeces; N.L. gen. n. *hominifaecis*, of human faeces)

Basonym: *Amedibacillus hominis* Abdugheni *et al*. 2023

The description is the same as that for *A. hominis* given by Abdugheni *et al*. [17]. The DNA G+C content of the type strain is 35 mol%. The 16S rRNA and genome sequence accession numbers are ON098491 and GCF_022487425.1, respectively. The type strain is NSJ-176^T^ (=CGMCC 1.17933^T^=KCTC 25355^T^), isolated from human faeces. The species is the later heterotypic synonym of *Hoskinsella hominis* New-5^T^ (=NSJ-61^T^=CGMCC 1.32837^T^=KCTC 15860^T^).

## Abbreviations

AAI: average amino acid identity
ANI: average nucleotide identity
blastn: Basic Local Alignment Search Tool nucleotide
CMB: chopped meat broth
dDDH: digital DNA–DNA hybridization
DMA: dimethyl acetal
GTDB: Genome Taxonomy Database
MALDI-TOF MS: matrix-assisted laser desorption/ionization-time-of-flight MS
MSA: multiple sequence alignment
MUSCLE: MUltiple Sequence Comparison by Log-Expectation
NCBI: National Center for Biotechnology Information
